# Genome Sequence of Environmental Isolate Staphylococcus aureus OS-6, Isolated from a Soil Sample

**DOI:** 10.1128/mra.00238-23

**Published:** 2023-05-22

**Authors:** Rajnish Prakash Singh, Kiran Kumari

**Affiliations:** a Department of Bioengineering and Biotechnology, Birla Institute of Technology, Ranchi, Jharkhand, India; DOE Joint Genome Institute

## Abstract

Staphylococcus aureus is a major opportunistic pathogen that causes infections in various animals, including humans. Here, we report a genome sequence of an environmental isolate, S. aureus OS-6. The genome consists of a circular 2,808,665-bp chromosome with a G+C content of 32.7%.

## ANNOUNCEMENT

Staphylococcus aureus is a well-known pathogen of many animal species including humans and is associated with community and nosocomial infections (1). The infections associated with S. aureus are regarded as one of the world’s leading causes of food consumption-related disease outbreaks (2). The selective pressure due to the presence of antibiotics or stress factors in the environment induced the development of antimicrobial resistance in staphylococci (3, 4). S. aureus generally colonizes human skin and mucous membranes, and approximately 30% of the general population will be colonized by S. aureus at any given time (5). However, little is known about the environmental strains of S. aureus.

Strain S. aureus OS-6 was isolated from a soil sample (clay soil) collected from Jhiri, Ranchi, India (23.40°N, 85.25°E), in the month of June 2022. We used nitrile gloves for the collection of soil, and it was brought to the lab in closed ziplock bags. The lab was fumigated before the bacterial isolation, and control LB agar plates were placed in the lab, parallel with our samples. On the control plate, there was no growth of any microorganisms.

One hundred microliters of serially diluted samples was spread on LB agar medium and kept for incubation at 37°C under static conditions. Based on the 16S rRNA sequencing analysis (6), one of the isolates, designated OS-6, was classified in the genus Staphylococcus. Here, we provide the genome sequence of S. aureus OS-6. A single colony of OS-6 was inoculated into 10 mL of LB broth medium and incubated at 37°C on a rotary shaker at 180 rpm overnight. Genomic DNA was extracted using the Qiagen DNA isolation kit, quality was tested using a spectrophotometer, and the DNA was further quantified using a Qubit 3.0 fluorometer (Thermo Scientific). The whole-genome sequencing was performed on a sequencing platform at Biokart Genomic using Illumina HiSeq, and the paired-end library was prepared by using the NEBNext Ultra DNA library prep kit. A total of 25.8 million reads were generated.

The reads were filtered to remove adapter sequences and low-quality sequences using the BBTools suite, version 38.95 (https://sourceforge.net/projects/bbmap/), and sequencing errors were corrected using Lighter version 1.1.2 (7). The Fast QC program was used for quality control of Illumina reads and BlobTools v.1.0 was used to check the presence of any contaminant DNA (8). The contig sequences obtained were polished using Pilon v.1.24 (9). The Illumina reads were assembled using Unicycler v0.4.8 genome assembly tools (10). The parameters used on Unicycler for assembling the raw sequences included minimum sequence length of 300 bp with minimum kmer fraction of 0.2, maximum kmer fraction of 0.95, minimum component size of 1,000, and minimum dead end size of 1,000 with no Pilon. CheckM v.1.2.2 was used to check the quality of the assembled genome sequence with a completeness of 99.7% and contamination of 0.3% (11). Default parameters were used for all software unless otherwise specified. Gene annotation of the generated chromosomal sequence was performed using the NCBI Prokaryotic Genome Annotation Pipeline (PGAP) (12). The genome assembly contained 35 contig sequences ranging from 340 bp to 923,976 bp in length. The draft genome consists of 2,808,665 bp, with an *N*_50_ value of 331,370 bp and a G+C content of 32.7%. The draft genome sequence contains a total of 2,773 protein-encoding genes, 81 pseudogenes, and 62 RNAs (3 rRNAs, 55 tRNAs, and 4 noncoding RNAs [ncRNAs]) as determined by the NCBI Prokaryotic Genome Annotation Pipeline (PGAP) v.6.2 (https://github.com/ncbi/pgap).

### Data availability.

The genome sequence also has been submitted to the NCBI under BioSample accession number SAMN33453448, BioProject accession number PRJNA938998, and SRA accession number SRR23625766. The draft genome sequences were submitted to the DDBJ under accession number JARESD000000000.
